# Exploring the Nature of Teachers’ Math-Gender Stereotypes: The Math-Gender Misconception Questionnaire

**DOI:** 10.3389/fpsyg.2022.820254

**Published:** 2022-04-14

**Authors:** Anna-Sophia Dersch, Anke Heyder, Alexander Eitel

**Affiliations:** ^1^Teaching and Learning With Media, Department of Educational Psychology, Institute for Psychology, Justus Liebig University Giessen, Giessen, Germany; ^2^Department of Educational and Differential Psychology, Institute for Psychology, TU Dortmund University, Dortmund, Germany

**Keywords:** stem education, misconceptions, questionnaire, teacher education, math-gender stereotypes

## Abstract

Stereotypes of girls having weaker mathematical abilities than boys (math-gender stereotypes) are one factor reducing women’s representation in mathematics. Teachers, as powerful socializers, often hold math-gender stereotypes. Reducing math-gender stereotypes in (student) teachers thus may foster women’s representation in mathematics. Yet knowing the stereotypes’ underlying assumptions is crucial to reducing it. Do math-gender stereotypes reflect elaborate, disproven theories about gender differences in math, meaning *math-gender misconceptions*? And if so, which math-gender misconceptions are behind math-gender stereotypes? This is the focus of the present research. The relevant literature implies the existence of three distinct misconceptions: (1) *empathizing-systemizing* (“As girls think rather empathically and boys think rather systematically, boys are on average more talented in math than girls”), (2) *girls’ compensation* (“To achieve equally good grades in mathematics, boys have to make less effort because they are more talented than girls are”), and (3) *girls’ non-compensability* (“Despite their on average stronger effort, girls are normally less proficient in math than boys”). We assessed these misconceptions in a student teacher sample (*N* = 303) using our newly developed *Math-Gender Misconceptions Questionnaire*. Our results offer support for the expected three-factor structure of math-gender misconceptions. All three math-gender misconceptions showed good to acceptable scale reliabilities. On average, preservice teachers did not hold (strong) math-gender misconceptions. But a subgroup of 48.2% of preservice teachers held at least one of the three misconceptions. The *empathizing-systemizing* misconception was the most prevalent (32.0%) among the three misconceptions. Descriptively, endorsing the math-gender stereotype correlated most strongly with the *empathizing-systemizing* (*r* = 0.43) and the *girls’ compensation misconception* (*r* = 0.44). This may indicate that especially these two misconceptions partly underlie math-gender stereotypes. As a consequence, refutation instructions designed to reduce these misconceptions may be a promising method to weaken math-gender stereotypes. Further research is needed to investigate to what degree reducing the present misconceptions is related to reducing math-gender stereotypes. Hence, this study is the first one of a planned series of studies on the relation between math-gender misconceptions and math-gender stereotypes.

## Introduction

Stereotypes of girls having weaker mathematical abilities than boys (math-gender stereotypes) are widely prevalent in Western societies (Nosek et al., 2010). Math-gender stereotypes reduce girls’ interest, motivation, and performance in math, and lead to women being less likely to pursue mathematical professions (e.g., Wang and Degol, 2017). Teachers, as powerful socializers, also endorse math-gender stereotypes (e.g., Gunderson et al., 2012). Reducing math-gender stereotypes in (student) teachers thus seems a promising way to foster the representation of girls and women in mathematics. However, to address these stereotypes effectively, we must know about their nature and underlying assumptions. Do math-gender stereotypes reflect elaborate, yet disproven, theories about gender differences in mathematical abilities, that is, *misconceptions* (e.g., Eitel et al., 2021)? And if so, which misconceptions about mathematical abilities exist? We aim to answer these questions in the present research. This is important, because such misconceptions do not dissipate on their own – instead, overcoming them requires specific instructions in teacher education (refutation texts; Eitel et al., 2019; Menz et al., 2021).

In the literature, we identified three potential misconceptions associated with math-gender stereotypes about mathematical abilities: First, boys are assumed to be inherently better in math, because they supposedly think more systematically than girls, whereas girls think more empathically (Baron-Cohen, 2005; for disprove, see Escovar et al., 2016). Secondly, girls are assumed to succeed as well as boys in math only because they are hardworking, whereas boys are simply talented. This belief was detected in teachers and other socializers (Tiedemann, 2002; Robinson-Cimpian et al., 2014; Sáinz et al., 2020). Thirdly, if mathematical abilities are perceived as fixed (for theories of *fixed* and *growth mindset* and their influence on learners, see Dweck, 1999; Gunderson et al., 2017) and girls are ascribed less mathematical talent, then girls would be unable to compensate for their poorer mathematical abilities. In this study, we developed the *Math-Gender Misconception Questionnaire* (*MGMQ*) to investigate to what degree these three potential misconceptions are empirically separable, present in a student teacher sample, and linked to related constructs such as fixed mindsets of math ability (e.g., Leslie et al., 2015). Note that this study is the first of a planned series of studies on the relation between math-gender misconceptions and math-gender stereotypes.

### Gender Stereotypes About Mathematical Abilities

There is evidence that girls’ – and boys’ mathematical abilities are not inherently different (Lachance and Mazzocco, 2006; Kersey et al., 2019). However, with age, math-gender differences favoring male students emerge in some countries (Else-Quest et al., 2010). These gender differences are relatively small compared to other performance differences (e.g., caused by economic status; Bloom et al., 2008). Further, such differences are usually found in older learners (e.g., Reilly et al., 2015) already influenced by societal gender attitudes (Eliot, 2010). Accordingly, gender differences are mediated by sex-role identity and related to cultural opportunity structures for women (Reilly, 2012). Moreover, gender stereotypes about girls’ and women’s lesser abilities in science, technology, engineering, and math (STEM) are widely prevalent in Western cultures (Nosek et al., 2010; Nosek and Smyth, 2011; Hand et al., 2017) and predict women’s lower STEM engagement (Hyde et al., 1990; Halpern et al., 2007; see Nosek and Smyth, 2011; for similar findings on reading and boys, see e.g., Retelsdorf et al., 2015; Muntoni and Retelsdorf, 2018). In this vein, in many Western countries, women remain underrepresented in the mathematical professions (Wang and Degol, 2017). The societal stereotypes of girls’ and women’s lesser math abilities (*math-gender stereotypes*) influence children from an early age (e.g., Eliot, 2010). Math-gender stereotypes are conveyed by parents, peers, and teachers (see e.g., Hannover, 2008). As school is especially important for children’s socialization (Wentzel, 2014), children are prone to being influenced by teachers’ math-gender stereotypes. According to the *Model of Achievement Related Choices* (Eccles et al., 1983), teachers, as part of the cultural milieu, hold gender stereotypes including math-gender stereotypes (Eccles, 2011; Gunderson et al., 2012). Teachers have more positive attitudes about male students’ math performance, overrate male students’ mathematical abilities and have higher expectations regarding male students’ mathematical success (Riegle-Crumb and Humphries, 2012; Robinson-Cimpian et al., 2014; for a literature review, see Li, 1999). Further, teachers attribute failure in math to a lack of talent among girls, but to a lack of effort among boys (Tiedemann, 2002), and demonstrate a gender bias when evaluating students’ performance in an experimental setting (underrating equal performance outcomes if they assume female learners achieved them (Avitzour et al., 2020; see also Holder and Kessels, 2017). Furthermore, the Eccles et al. (1983) model proposes that teachers’ beliefs and behaviors influence their students’ own gender roles and stereotypes (see Eccles, 2011). Teachers’ own math-gender stereotypes thus predict students’ math-gender stereotypes (Keller, 2001). Although these math-gender stereotypes seem to have decreased in school children (e.g., Passolunghi et al., 2014), recent research still suggests that even primary school children hold the perception of math being male-typed (Miller et al., 2015). These stereotypes then influence students’ expectation of success and subjective task value (e.g., in mathematics), which in turn influences students’ achievement-related choices. Math-gender stereotypes of girls lead to girls tending to make academic choices against mathematics (see Eccles, 2011). Apart from academic choices, math-gender stereotypes influence girls’ sense of identity. The idea of math being male-typed (Miller et al., 2015) leads to girls developing less interest or preference for math when forming their identity. Thus, girls do not engage further with math, as girls try to establish their identity as distinct from the boys’ identity and from male-typed interests (Bian et al., 2017). All in all, math-gender stereotypes reduce girls’ interest, motivation, and performance in math, and, ultimately, lead to women being less likely to pursue mathematical professions (e.g., Wang and Degol, 2017). Further, according to learning theories, girls (and boys) learn to behave according to gender stereotypes because parents, *teachers* and peers reinforce them for doing so (Mischel, 1966; Hannover, 2008). This process of operant conditioning leads to girls’ engaging less with math as teachers – due to their math-gender stereotype – reinforce girls less than they reinforce boys for engaging with math. Besides that, math-gender stereotypes influence girls’ – and women’s performance through social-psychological mechanisms such as *self-fulfilling prophecies* or *stereotype threat*. When societal stereotypes are activated, girls are more likely to behave in a way that fulfills societal stereotypes and expectations. For example, teachers implicitly expressing their math-gender stereotypes and thus treating girls differently may instigate a worse math performance [see *self-fulfilling prophecy* (Merton and Merton, 1968)]. Just the fear itself of negative judgment in light of the math-gender stereotype can cause a disruption leading to girls’ performing worse in math [see *stereotype threat* (Steele and Aronson, 1995)]. This means that teachers, who – because of their math-gender stereotypes – expect girls to perform worse, in fact contribute to female learners actually performing worse in standardized math tests (Geis, 1993; Smith et al., 1999; Spencer et al., 1999).

Finally, as powerful socializers, teachers do not only endorse math-gender stereotypes, their math-gender stereotypes influence girls’ math attitudes and performance negatively (Gunderson et al., 2012; Carlana, 2019). Reducing teachers’ stereotypes may therefore represent a means to increase women’s representation in mathematics. To weaken stereotypes, however, it is important to know about their nature and underlying assumptions, which is in the focus of this research.

### Interrelation of Math-Gender Stereotypes and Math-Gender Misconceptions

Math-gender stereotypes and misconceptions about math abilities based on gender (*math-gender misconceptions*) are two theoretically related but separable constructs.

Stereotypes are based on oversimplified, overgeneralized beliefs (Klineberg, 1951); for instance, beliefs that a certain group member has certain attributes because they are a member of a group (Greenwald et al., 2002). Thus, the math-gender stereotype is the over-simplified, overgeneralized belief of girls having weaker mathematical abilities because of their gender (Math-gender). Stereotypes are rarely fully refuted (FitzGerald et al., 2019; Kollmayer et al., 2020). This may be the case, because the specific reasoning or (mis-)conceptions behind a global stereotype are hard to grasp and therefore hard to target (e.g., by refutation texts; Tippett, 2010). Likewise, empirical evidence showing that math-gender stereotypes persist despite being incorrect (e.g., Gunderson et al., 2012) is paralleled by the scarcity of research on how instruction can overcome these math-gender stereotypes (Kollmayer et al., 2020). In this study, we want to explore the specific reasoning behind teachers’ math-gender stereotypes to prospectively provide refutation instruction. More specifically, we want to know whether endorsing math-gender stereotypes is related to holding *math-gender misconceptions* – subjectively plausible, yet disproven, theories about gender differences in mathematical abilities (for misconception definition, see Vosniadou, 1994; Chi and Roscoe, 2002; Hughes et al., 2013).

### Math-Gender Misconceptions

Previous research suggests the potential presence of *three* specific misconceptions underlying gender stereotypes about mathematical abilities.

The first potential misconception refers to the Empathizing-Systemizing theory (Baron-Cohen, 2005) to explain the assumption of boys’ better inherent mathematical abilities compared to girls’ inherent mathematical abilities. The prominent Empathizing-Systemizing theory assumes that biological determinants explain gender differences in math. The Empathizing-Systemizing theory states that, because pre-natal testosterone-exposure is higher in the male fetus than the female, boys develop more systematic thinking in relation to less empathic thinking. Because pre-natal testosterone-exposure is lower in girls than boys, girls develop less systematic thinking in relation to more empathic thinking. According to the Empathizing-Systemizing theory, girls’ weaker systematic thinking leads to lower mathematical abilities (Baron-Cohen, 2005). This view, however, is very one-sided and excludes societal factors scientifically proven to be important (e.g., Hannover, 2008; Eliot, 2010; Eccles, 2011; Wang and Degol, 2017). Further, even though female participants in some research did exhibit a higher ratio of empathic to systematic thinking than did men and vice-versa (e.g., Greenberg et al., 2018), this ratio-difference did not predict mathematical performance, even when researched in a huge sample (Escovar et al., 2016). In addition, the idea of empathic thinking being negatively associated with systematic thinking is not very convincing, considering that both refer to the construct of general thinking abilities [general intelligence (g); Gottfredson, 1998]. Consequently, the Empathizing-Systemizing theory itself represents a math-gender misconception (*empathizing-systemizing* misconception).

The second potential misconception, termed *girls’ compensation*, refers to the belief that girls achieve similar math results as boys because they are hardworking, whereas boys are simply talented. However, girls actually report *less* intrinsic motivation in math than boys (e.g., Skaalvik and Rankin, 1994; Rodriguez et al., 2020; Heyder et al., 2020). As motivation is a strong predictor for effort and persistence (Skaalvik et al., 2015), girls are likely to be less driven to succeed in math. Girls are therefore very unlikely to achieve similar math results as boys only because they work harder. Furthermore, results from various studies suggest a similar level of mathematical talent in boys and girls: At a young age, girls and boys reveal gender similarities – rather than differences – in neural functioning when engaging with mathematical content (Kersey et al., 2019). In a longitudinal observation of primary school children (Lachance and Mazzocco, 2006), sex differences in math performance measured via standardized tests were minimal to non-existent. These empirical results offer no support for the idea that girls have lower math abilities overall. *Girls’ compensation* thus counts as a math-gender misconception.

The third potential misconception, termed *girls’ non-compensability*, also refers to the belief about gender differences in mathematical talent. However, here the focus is on innate differences in mathematical talent that girls cannot compensate for later in life, because talent is assumed to be fixed. This *fixed mindset* is especially common in mathematics and other STEM subjects (e.g., Leslie et al., 2015; Gunderson et al., 2017; Canning et al., 2019) and also identified among teachers (Heyder et al., 2020). A fixed mindset stands in opposition to evidence of educational achievement, such as the *growth mindset* proposed by Dweck (1999, 2015). Accordingly, rather than being fixed, skills can improve over time with practice. However, people who hold the *girls’ non-compensability* misconception assume that talent is fixed, and simultaneously ascribe girls less mathematical talent. In so doing, they assume girls cannot compensate for inherent talent differences in mathematical abilities. However, as described before, there is no evidence supporting the idea of girls having lower innate math abilities. Furthermore, the combination of a fixed mindset and lack-of-talent assumptions is especially detrimental for female students’ math-attitudes (Dweck, 2015; Heyder et al., 2019, 2020; Muenks et al., 2020) and for their performance (Canning et al., 2021).

### Current Study and Hypotheses

In this study, we present the newly developed *Math-Gender Misconception Questionnaire (MGMQ*) to assess teachers’ misconceptions about gender differences in mathematics abilities. These misconceptions may underlie stereotypical thinking and behavior (see section “Interrelation of Math-Gender Stereotypes and Math-Gender Misconceptions”). By means of this questionnaire, we investigated to what degree the three potential misconceptions (*empathizing-systemizing*, *girls’ compensation*, *girls’ non-compensability*) are (1) empirically separable (*structure hypothesis*) and measurable by reliable scales, (2) present in a student teacher sample (*prevalence hypothesis*), and (3) linked to theoretically related constructs (*association hypothesis*).

#### Structure Hypothesis

We expect the MGMQ to assess three empirically separable, yet positively interrelated misconceptions. All three of the previously described misconceptions (see section “Interrelation of Math-Gender Stereotypes and Math-Gender Misconceptions”) are related to beliefs about gender differences in mathematical talent. Nevertheless, each misconception focuses on a different aspect: The *empathizing-systemizing* misconception provides an over-simplified explanation for the existence of gender differences in mathematical talent. The *girls’ compensation* misconception refers to girls managing to compensate for their lesser mathematical talent by investing effort. The misconception of *girls’ non-compensability* puts girls’ un-ability to compensate for their lack of talent into focus. Therefore, we expected the MGMQ data to fit a three-factor structure of math-gender misconceptions better than a general-factor structure with one homogeneous misconception construct in a confirmatory factor analysis.

#### Prevalence Hypothesis

We expect student teachers to rather endorse the first two of the three potential misconceptions. Given the high prominence and face validity of the idea that girls think more empathically whereas boys think more systematically (Empathizing-Systemizing theory; Baron-Cohen, 2005), some student teachers may also believe that these thinking differences are related to worse mathematical abilities – a misconception (*empathizing-systemizing* misconception). Further, we expect some student teachers to endorse the *girls’ compensation* misconception referring to the belief that girls only succeed in math because they work hard, whereas boys who succeed are talented. This belief is likely to exist among teachers, because teachers attribute girls’ better math grades than boys’ math grades to the girls’ greater effort (Sáinz et al., 2020). Further, teachers perceive girls only as similarly math-competent as boys if girls work harder (Robinson-Cimpian et al., 2014). Likewise, teachers attribute girls’ weak mathematical performance to lacking talent, and boys’ weak mathematical performance to lacking effort (Tiedemann, 2002). This research also suggests that (student) teachers may endorse the *girls’ non-compensability* misconception to a lesser degree than the *girls’ compensation* misconception.

#### Association Hypothesis

We first expect the three math-gender misconceptions to relate positively with the common math-gender stereotype found in previous research using a simple *female-to-male*-rating for math (for a similar measure, see Nosek, 2007, Nosek et al., 2010). We expect this association, as there are similarities and overlaps amongst math-gender stereotypes and math-gender misconceptions (Klineberg, 1951; Chi and Roscoe, 2002; Kollmayer et al., 2020). More specifically, we expect math-gender stereotypes to be partly based on math-gender misconceptions, which should be expressed in a moderate to high correlation between the two. Secondly, we expect that holding the girls’ non-compensability misconception will relate positively with holding fixed-ability mindsets for mathematics (Leslie et al., 2015). Holding the girls’ non-compensability misconception means assuming that girls’ lack of talent cannot be compensated for, and is thus fixed. This misconception is similar to the idea of fixed ability mindsets for mathematics.

## Materials and Methods

### Participants and Recruiting

A total of 303 student teachers [242 women, 61 men, *M*_age_ = 21.73 (*SD* = 4.7, range = 18–51 years)] completed our online survey without dropping out. These data sets were complete (no missing data amongst them). The student teachers had studied on average for 2.28 semesters (*SD* = 2.28, range = 2–16 semesters). The student teachers’ school subjects were mostly German (*n* = 146) and math (*n* = 118), followed by other common subjects (e.g., English, biology, politics and economics, philosophy, geography, languages such as French, Spanish, or Latin). More than half of the participants (168; 55.5%) studied at least one STEM subject. Participants were studying to teach at the elementary (*n* = 79) or secondary school level (*n* = 191). Some participants were studying to teach in vocational education (*n* = 7) or special needs education (*n* = 63). Participants from all over Germany took part in this study; most were from Hessen. The participants, on average, held positive views about gender equality and feminism (*M* = 3.61, *SD* = 0.84; scale of 1 = not at all to 5 = very).

The communicated topic of the study was “Mathematics and Gender.” The online survey completion was possible between May and July of 2021. We recruited participants via teacher education lectures and seminaries as well as via acquaintances. In total, 360 people clicked on the survey link, of which 303 participants (84.2%) completed the survey. Two people declined consent; the other 55 participants (15.3%) dropped out during the study and were not included in our analyses, yielding the final sample of 303 student teachers.

### Study Instruments

#### Math-Gender Misconception Questionnaire

**FIGURE 1:**
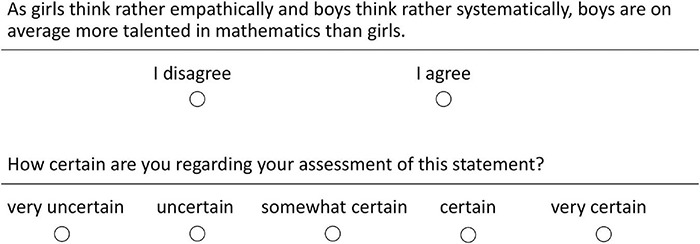
Example item.

The self-developed Math-Gender Misconceptions Questionnaire (MGMQ; see Table 1 for an English translation of the misconception items and Supplementary Appendix A for the German original containing all items) served as our main study instrument. It consisted of 30 items. These items comprised statements that participants first must answer with “I disagree” or “I agree” (i.e., verification). Second, each statement comprised a five-point Likert-scale assessing the participants’ certainty of having correctly responded to the current statement. The answer options were *very certain*, *certain*, s*omewhat certain*, *uncertain*, *very uncertain* (i.e., certainty rating). Certainty ratings were horizontally aligned and presented below the corresponding verification part (see Figure 1, for an example item). These two ratings per item are crucial for assessing misconceptions: Holding a misconception should reflect in *incorrect* answers made with a (relatively) *high certainty*. Incorrect answers with low certainty would rather reflect missing conceptions (see Eitel et al., 2021, for the argumentation). Of the 30 items in the MGMQ, 15 items targeted math-gender misconceptions (see Table 1) and 15 items were filler items. Of the 15 misconception items, always five referred to each of the three hypothesized misconceptions (*empathizing-systemizing*, *girls’ compensation*, *girls’ non-compensability*). The correct answer was to disagree with the misconception items.

**TABLE 1:** Descriptive values for misconception items and scale reliabilities of the MGMQ.

Empathizing-systemizing (ES): ω = 0.88; asymptotic ω = 0.90	Agreement rates (min. = 0, max. = 1)	Response certainty (min. = 0, max. = 4)	Misconception score*^a^* (min. = −4, max. = +4)	Item-total correlation (min. = 0, max. = 1)
ES1: *As girls think rather empathically and boys think rather systematically, boys are on average more talented in math than girls*	0.32	2.50 (0.94)	−1.30 (2.33)	0.57
ES2: *Mathematical relationships are usually easier to understand for boys than girls, because boys think in more systematic contexts*	0.39	2.31 (0.92)	−0.65 (2.40)	0.74
ES3: *As boy, more likely think in systematic categories, they fulfill more cognitive prerequisites for math than girls do*	0.39	2.22 (1.00)	−0.73 (2.33)	0.75
ES4: *Female empathy makes it easier for girls to deal with people, while boys are usually more gifted in systematic thinking and thus in math*	0.49	2.50 (0.97)	−0.28 (2.67)	0.77
ES5*: On average, girls think more empathically than boys do, while boys are more talented in systematic thinking and thus also in math*	0.44	2.34 (0.99)	−0.53 (2.49)	0.81

**Girls’ compensation (GC):** ω = 0.76; asymptotic ω = 0.91				
GC1: *Mathematical content often comes easily to boys, while girls on average have to make more effort*	0.14	2.76 (0.86)	−2.16 (1.93)	0.58
GC2: *Girls normally have to work harder to perform as well in math as boys*	0.23	2.63 (0.83)	−1.60 (2.25)	0.61
GC3: *Girls compensate for their usually less aptitude in math compared to boys by being more diligent*	0.48	2.36 (0.91)	−0.19 (2.52)	0.46
GC4: *Girls usually need additional help to perform on par with boys in math*	0.14	2.61 (0.98)	−1.96 (1.98)	0.54
GC5: *To achieve equally good grades in math, boys have to make less effort because they are more talented than girls are*	0.17	2.67 (0.98)	−1.97 (2.05)	0.71

**Girls’ non-compensability (GN):** ω = 0.72; asymptotic ω = 0.68				
GN1: *Since girls are on average less mathematically gifted, they should be assessed with different criteria than boys*	0.05	3.34 (0.87)	−3.10 (1.53)	0.56
GN2: *Girls should be rewarded with good grades for their stronger efforts in math, as they are not naturally as good at math as boys*	0.08	3.08 (0.98)	−2.74 (1.71)	0.62
GN3: *If the top of the class in math is a boy, it is because, in addition to his effort, he possesses a natural talent in math that diligent girls often lack*	0.18	2.80 (1.01)	−2.08 (2.14)	0.47
GN4: *Girls cannot fully compensate for their lack of aptitude for math with their on average greater diligence*	0.14	2.72 (0.89)	−2.11 (1.93)	0.45
GN5: *Despite their on average stronger effort, girls are normally less proficient in math than boys*	0.21	2.56 (0.97)	−1.67 (2.17)	0.43

**All items:** ω = 0.82; asymptotic ω = 0.69				

*Agreement rates represent the proportion of participants agreeing statement. Descriptive values for response certainty and misconception scores represent means and standard deviations (in parentheses).*

*^a^Calculated by converting agreement into +1 and disagreement into –1, then multiplied with response certainty.*

The misconception items asked for all of the characterizing aspects of each hypothesized math-gender misconception, by also referring to research findings (Dweck, 1999; Tiedemann, 2002; Muenks et al., 2020; Sáinz et al., 2020), and academic as well as non-academic resources (Baron-Cohen, 2005; Escovar et al., 2016). For the *empathizing-systemizing* misconception, we constructed each of the items to address the combination of the following two stereotypical beliefs: (1) boys are better at math than girls (2) because boys think more systematically, whereas girls think more empathically. We constructed such complex items because only the combination of the two stereotypical beliefs [(1) gender differences in empathizing-systemizing and (2) their direct relation to mathematics performance] is a misconception. An example item was “As girls think rather empathically and boys think rather systematically, boys are on average more talented in mathematics than girls.” The same rationale for constructing items applies to the two other misconceptions.

For the *girls’ compensation* misconception, items focused both on (1) the belief about gender differences in math talent and on (2) the beliefs that either girls compensate for their fewer talent through hard work or teachers compensate for girls’ fewer talent by treating them differently than boys (e.g., more support). An example item regarding *girls’ compensation* was “To achieve equally good grades in math, boys have to make less effort because they are more talented than girls are.”

For the *girls’ non-compensability* misconception, items focused on both (1) the belief about girls being unable to compensate for their lack of talent even with hard work and (2) the belief about implications of this non-compensability in the treatment of genders (such as grading the girls more generously). An example item regarding *girls’ non-compensability* was “Despite their on average stronger effort, girls are normally less proficient in math than boys.”

We intentionally formulated the misconception items as false statements to gain direct information as to whether the student teachers endorsed this particular misconception. Specifically, disagreeing with a correct statement (“the Earth is a sphere”) does not give direct information regarding the underlying misconception (the Earth could be flat, rectangular, a semi-sphere, etc.), whereas agreeing with the incorrect statement (“the Earth is flat”) provides direct information about endorsing this particular misconception (cf. Eitel et al., 2021).

The remaining 15 filler-items described true statements related to the math-gender gap, thus they were not misconceptions. An example filler item was “Amongst girls, math is more disliked than amongst boys.” The correct answer was to agree with these filler items. The filler-items served to balance the questionnaire. In total, 50% of the statements in the questionnaire were true (i.e., filler-items), while the other half of statements was untrue (i.e., misconception-items). We balanced the questionnaire in order to minimize response biases in the form of acquiescence tendencies (Moosbrugger and Kelava, 2012) because participants might think “some statements must be true” and answer accordingly (cf. Eitel et al., 2021).

Prior to inclusion in the questionnaire, an expert on the math-gender gap and an expert on developing questionnaires revised all items. Additionally, a four-member expert panel (one professor, two postdoctoral researchers, and a Ph.D. student from educational psychology) discussed and refined the questionnaire. Furthermore, we evaluated a prior version of this questionnaire within a pilot study with 246 student teachers. Results of this pilot study suggested that not one unitary construct of math-gender misconceptions but three misconceptions scales might best explain the questionnaire responses, namely the scales of *empathizing-systemizing*, *girls’ compensation*, and *girls’ non-compensability*. Based on these preliminary findings, we constructed the MGMQ with 15 misconception items, as the former version did not have sufficient misconception items per scale.

#### Other Instruments

Furthermore, we assessed math-gender stereotypes similar to previous research (Nosek et al., 2010) by asking participants to indicate whether they perceived math as female or male. We used only part of the measure applied by Nosek et al. (2010), who assessed implicit and explicit math-gender stereotypes together with liberal arts-gender stereotypes. Additionally, we extended the scale range to 9 answer options, starting from 1 (“very female”) via 5 (“neutral”) to 9 (“very male”), to potentially increase variance. The results nevertheless revealed that answers of 1 (“very female”), 8 and 9 (“very male”) were outliers in the answer distribution. We thus winsorized the distribution to reduce the biasing effect of the outliers in the correlational analyses.

We then assessed participants’ feminism using three items with five-tier Likert-scales each (from *not at all* to *very*). An example item was: “How important is the equality of the genders to you?” The internal consistency of the scale was good (ω = 0.79). For all three items in German and English, see Supplementary Appendix B.

We also assessed teachers’ *fixed mindset about math ability* with two items adapted from Leslie et al. (2015) and Heyder et al. (2020). An example item was: “Being among the best in math requires a special aptitude that just cannot be taught.” Both items were highly correlated (*r* = 0.66, *p* < 0.001) so that we calculated the mean score of both items (*M* = 4.07, *SD* = 1.42).

Before ending the study, participants filled in their demographics such as age, sex, gender, mother language, study subjects, school type they will teach at or already teach at, and semesters studied.

### Procedure

When clicking on the web link, participants initially read about the voluntary nature of their participation, that they could end the study whenever they wanted without facing disadvantages, and that we would store all data for 10 years anonymously for the purpose of research only. Participants then gave their informed consent. Participants then read the instruction for the misconception questionnaire, which they then filled in. Then, participants rated how they perceived mathematics on a 9-tier Likert-scale (*female* to *male*). Afterward, participants filled in two items each on fixed mindset in math. They also indicated their attitude toward feminism. Participants then provided basic demographic information. After participation, we thanked the participants and provided a full debriefing text. Participants took on average 13:44 min (*SD* = 5:48 min) to complete the survey.

### Scoring the Misconceptions

We calculated misconception scores by multiplying agreement (coded with +1)/disagreement (coded with −1) and response certainty (coded from 0 = *very uncertain* to 4 = *very certain*; see Eitel et al., 2021). Thereby, we accounted for the nature of misconceptions: Misconceptions are incorrect and are subjectively highly plausible. Thus, if the person assumes an incorrect statement to be more plausible, this person endorses that statement more strongly, reflecting in higher certainty (see Eitel et al., 2021). This stronger endorsement of a misconception is reflected in higher misconception scores (see Table 1, for descriptive values). Participants who were very uncertain about an answer (coded with 0), regardless of whether it was correct or not (±1), got a misconception score of 0 (i.e., ±1 × 0 = 0), because their (dis-)agreement was probably guessing and indicated no misconception (see Eitel et al., 2021). The stronger participants believed in the misconception, the more certain participants were in their agreement with a false statement (e.g., scores of 2 vs. 4 in the certainty rating). Accordingly, a stronger misconception was indicated by a higher misconception score (e.g., 2 vs. 4). Using this multiplication method, the range of possible values per item was −4 to +4, making it possible to approximate the level of interval-scaled data required to perform confirmatory factor analyses with (robust) maximum likelihood estimation (see Eitel et al., 2021).

We assumed a misconception to be prevalent, whenever participants answered at least one of the five items per misconception scale *incorrectly with high certainty* (i.e., response certainty of 3 or higher, on scale from 0 to 4; see previous section). We did so because a *mixed (mis-)conception* would be prevalent in that case (see Vosniadou, 1994). Misconceptions can be very extreme (“The earth is flat”), but they can also be “alleviated” by integrating correct information (“The earth is round”). However, this alleviation may lead to a so-called *mixed* misconception (“The earth is round, but where we stand on it, it must be flat for us not to fall off”). This would still require further refutation (Vosniadou, 1994). One incorrect answer per misconception scale (made with high certainty) already indicates such a (mixed) misconception, which requires refutation in order to achieve a correct conception (Vosniadou, 1994; see Dersch et al., 2022).

### Data Analysis

We used IBM SPSS statistics^®^ for data preprocessing and item statistics. We used R for statistical computing (R Core Team, 2017; version 3.6.23) with the *psych* package for reliability analyses (Revelle and Condon, 2019). We calculated McDonald’s omega (ω) for robust reliability estimation even when item-scale correlations are not tau-equivalent (Deng and Chan, 2017). Asymptotic omega simulates the theoretical omega obtained for a test of infinite length with a structure similar to the observed test. Modest reliability for McDonald’s omega is at around 0.70 (Nunnally, 1978). However, this convention should be considered with some caution as satisfactory values depend on the measurement purpose (e.g., group statistics or individual assessment) and on the nature of the scale. If assessing broad or heterogeneous constructs, even relatively low coefficients of criterion reliability (e.g., 0.50) do not seriously attenuate validity coefficients (Schmitt, 1996).

We used the *lavaan* package for confirmatory factor analysis (Rosseel, 2012) to inspect the internal structure of the MGMQ by estimating its construct validity. We used maximum likelihood estimation with robust standard errors (MLR) to handle our interval data with moderate deviations from the normal distribution (Li, 2016). We considered the global model fit to be sufficiently good if the following criteria were met: a CFI (comparative fit index) value equal to or higher than 0.95, a root mean square error of approximation (RMSEA) smaller than 0.06 (Hu and Bentler, 1998), and an standardized root mean square residual (SRMR) smaller than or equal to 0.07 (Yu, 2002). We considered the local model fit to be acceptable if values for the fully standardized factor loadings were statistically significant (*p* < 0.05) and higher than 0.30 (Nunnally, 1978; Cristobal et al., 2007).

## Results

### Structure Hypothesis

**FIGURE 2:**
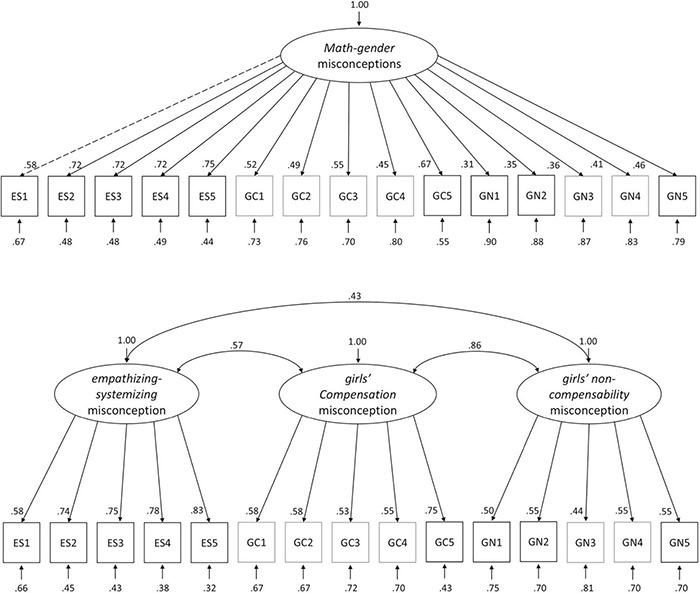
General factor model vs. three-factor model. The three-factor model fit better than the one-factor model.

We first examined the MGMQ’s factorial structure by comparing global and local fit measures of two structural models against each other in a confirmatory factor analysis. We expected the MGMQ data to better fit a correlated three-factor model of math-gender misconceptions (*empathizing-systemizing, girls’ compensation*, and *girls’ non-compensability*) than a general-factor model with one misconception construct. Accordingly, results revealed an overall acceptable global fit for the three-factor model (with five items per factor), *CFI* = 0.94, *RMSEA* = 0.058, *SRMR* = 0.057, χ^2^ = 157.75, *df* = 87, *p* < 0.001. The factors *girls’ compensation* and – *non-compensability* were highly positively correlated to each other (*r* = 0.86, *p* < 0.001), and to *empathizing-systemizing* (*r* = 0.72, *p* < 0.001; *r* = 0.51, *p* < 0.001). Results revealed an unacceptable global fit for the general-factor model, *CFI* = 0.80, *RMSEA* = 0.10, *SRMR* = 0.08, χ^2^ = 296.98, *df* = 90, *p* < 0.001. Supporting the structure hypothesis, the model fit of the three-factor model was statistically significantly better than the fit of the general-factor model, χ^2^(3) = 62.50, *p* < 0.001. On the level of local model fit, factor loadings were all significant (all *p*s < 0.01) and ranged between 0.44 and 0.83 for the three-factor model (*M* = 0.62, *SD* = 0.12; see Figure 2). Scale reliabilities [using McDonald’s omega (ω)] were good for *empathizing-systemizing* (ω = 0.88), acceptable for *girls’ compensation* (ω = 0.76), and acceptable for *girls’ non-compensability* (ω = 0.72).

### Prevalence Hypothesis

We expected student teachers to rather endorse the first two of the three gender misconceptions about mathematical abilities. As expected, more student teachers believed that boys are inherently better in mathematics because they think more systematically (*empathizing-systemizing*; 32.0%), and that girls are only as good in mathematics as boys because they work harder (*girls’ compensation*; 26.7%) and that girls cannot compensate for their lower mathematical abilities (*girls’ non-compensability*; 17.5%). Overall, 14.2% of student teachers endorsed both the *empathizing-systemizing* and the *girls’ compensation* misconception, whereas 44.6% of student teachers endorsed at least one of these two misconceptions. In total, 48.2% of student teachers endorsed at least one of the three misconceptions. However, on average, student teachers had negative misconception values in the MGMQ (see Table 1). This indicates that the majority of student teachers – correctly – disagreed with the misconception items and did not hold (strong) math-gender misconceptions.

### Association Hypothesis

We expected math-gender misconceptions to be positively associated with the prevalence of math-gender stereotypes. We found that 141 out of 303 student teachers indicated math to be more male than female, yielding a prevalence rate of 46.5%. A total of 150 student teachers (49.5%) indicated math to be equally male and female, whereas only 12 student teachers (4.0%) indicated math to be more female than male. Overall, the latent correlation between math-gender misconceptions and holding the math-gender stereotype was moderate, *r* = 0.45, *p* < 0.001. Descriptively, we found that the *empathizing-systemizing*, *r* = 0.43, *p* < 0.001, and the *girls’ compensation* misconception, *r* = 0.44, *p* < 0.001, correlated stronger with holding the math-gender stereotype than the *girls’ non-compensability* misconception, *r* = 0.25, *p* = 0.01.

Apart from that, we expected holding a fixed ability mindset for mathematics (Dweck, 1999; Leslie et al., 2015) to correlate more positively with the *girls’ non-compensability* than with the *girls’ compensation* misconception. We found that student teachers with a stronger fixed ability mindset for mathematics believed more strongly in all three misconceptions (*r* = 0.28, *p* < 0.001), however, not to a stronger degree in the *girls’ non-compensability* misconception (*r* = 0.22, *p* = 0.004) than in the *girls’ compensation* misconception (*r* = 0.28, *p* < 0.001).

## Discussion

Math-gender stereotypes held by important socializers like teachers may be contributing to the underrepresentation of girls and women in STEM (for a review, see Gunderson et al., 2012). The goal of this research was to explore the *specific* misconceptions underlying math-gender stereotypes in a student teacher sample. To this end, we first analyzed the structure and prevalence of three potential misconceptions using the newly developed Math Gender Misconceptions Questionnaire (MGMQ). Afterward, we inspected to what degree holding these misconceptions related to holding math-gender stereotypes, and fixed mindsets about math ability.

### Structure of Math-Gender Misconceptions Amongst Preservice Teachers

We constructed the MGMQ to uncover a three-factor structure of misconceptions about gender differences in mathematical abilities that we expected to observe based on prior research: *empathizing-systemizing*, *girls’ compensation* and *girls’ non-compensability*. We obtained evidence for the supposed tree-factor structure via confirmatory factor analysis. The three-factor model fit the data better than the model assuming one general misconception factor (see Figure 2). Math-gender misconceptions are thus expressed through three distinct factors. (1) There is the *empathizing-systemizing* misconception assuming that pre-natal testosterone-exposure levels are lower in girls than in boys, which leads to girls thinking less systematically in relation to more empathically. Girls’ less systematic thinking – according to this misconception – leads to girls’ lower mathematical abilities (Baron-Cohen, 2005). (2) The *girls’ compensation* misconception assume that girls are more hardworking than boys, resulting in their equally good performance in math (e.g., equal grades; Tiedemann, 2002; Sáinz et al., 2020). (3) The *girls’ non-compensability* misconception assumes that girls are not only less talented in math – for example due to the *empathizing-systemizing* misconception – but furthermore, they lack the means to compensate for their disadvantage, as math talent is fixed (Dweck, 1999; Leslie et al., 2015).

The *empathizing-systemizing* scale showed good reliability; all items correlated substantially with the construct (see Table 1). The *girls’ compensation* and - *non-compensability* scales showed acceptable reliabilities. The higher reliability of the *empathizing-systemizing* scale, compared to the other two scales, may be due to the items of *empathizing-systemizing* being very homogeneous; they all referred to the explanation of talent differences in boys and girls in mathematics. Items on the other two scales referred to both the talent differences in boys and girls in mathematics and the consequences of such talent differences. Items on the *girls’ compensation* scale refer to (1) girls having less talent in mathematics, and (2) girls usually compensating for their lesser talent. Items on the *girls’ non-compensability* scale refer to (1) girls having less talent in mathematics, and (2) how girls should be treated to adapt to their lack of talent (lower standards for girls; see Table 1, for an overview of all items). Meaning, *girls’ compensation* as well as *girls’ non-compensability* are broader and more heterogeneous constructs, which may explain their lower reliability coefficients than for the *empathizing-systemizing* scale.

### Prevalence and Correlates of Math-Gender Misconceptions

Almost half of the preservice teachers (48.2%) held at least one of the three misconceptions. A majority of student teachers, however, held no math-gender misconceptions, even according to the strict criteria we applied. This finding led to negative average math-gender misconception scores among student teachers in this sample (see Table 1), which imply that on average, math-gender misconceptions are not (strongly) prevalent. These results are encouraging, even if they are still far from ideal. The prevalence of math-gender misconceptions among a subgroup of student teachers is still worrying, since even endorsing just one the misconceptions can affect teachers’ instruction. As a consequence, misconceptions may cause a different treatment of the genders (e.g., Carlana, 2019), and reinforce math-gender stereotypes among schoolchildren (e.g., Geis, 1993; Eccles, 2011; Gunderson et al., 2012). The math-gender stereotypes weaken female representation in mathematical careers (e.g., Eccles, 2011; Wang and Degol, 2017). As teachers function as multipliers of their own knowledge and beliefs and teach many students during their career, misconceptions deserve attention and interventions in teacher education and training, even if only a subgroup of teachers seems to endorse such misconceptions.

As expected, both the *empathizing-systemizing* (32.0%) and *girls’ compensation* misconception (26.7%) seemed to be more prevalent than the *girls’ non-compensability* (17.5%) misconception. This difference in prevalence may partially be due to social desirability. Agreeing to the *empathizing-systemizing* misconception may be more socially desirable than agreeing to statements on the two other misconception scales, because the former statements (1) highlight girls’ empathic and social abilities and (2) provide an explanation for girls’ lack of talent that did not blame the girls themselves, but rather their genes or pre-natal influences on their body. Like for benevolent sexism (Glick and Fiske, 1996, 1997) these two apparently “positive” beliefs about girls might have been more socially acceptable than agreeing with the beliefs captured by the other two misconceptions.

The other two misconceptions consisted of statements displaying obvious, less benevolent sexism, such as indicating that (1) girls lack talent and (2) the genders should be treated differently and thus unequally. Such attitudes tend to be rejected nowadays among well-educated students in Western societies, like those in our study sample: Accordingly, the students in our sample indicated moderate to high agreement with feminism, which correlated negatively with misconception endorsement (*r* = −0.21, *p* = 0.001). This lower social desirability thus may have reduced agreement rates with the *girls’ compensation* and *girls’ non-compensability* scale, even though actual beliefs may differ from what participants indicated. Furthermore, the awareness that fixed mindsets in teachers are detrimental to their students (e.g., Canning et al., 2019, 2021; Heyder et al., 2020) seems to be increasing in (teacher) education (Dweck, 2016). Thus, especially the *girls’ non-compensability* scale – theoretically a combination of fixed ability mindset ideas and promoting girls’ lesser abilities, might be perceived as socially undesirable, which could have contributed to the (relatively speaking), lowest endorsement rates.

Furthermore, the significant correlation between fixed mindset in math and the *girls’ non-compensability* misconception as well as the non-significant correlation with the *girls’ compensation* misconception supports the construct validity of the MGMQ’s constructs: It is only when abilities are perceived as fixed that there is no way to compensate for low abilities. Since fixed mindsets in math have been found to be detrimental only in terms of female students’ intrinsic motivation and ability self-concepts (Heyder et al., 2020), these associations further corroborate the importance of the *girls’ non-compensability* misconception for female students’ engagement in math.

Our findings also support prior research findings of (preservice) teachers holding explicit math-gender stereotypes (e.g., Li, 1999; Tiedemann, 2002; Cimpian et al., 2016; Sáinz et al., 2020). Also in our study, about half (49.5%) of the preservice teachers held explicit math-gender stereotypes. These explicit math-gender stereotypes were associated with math-gender-misconceptions to a moderate degree (*r* = 0.45), tentatively supporting the idea of math-gender-misconceptions underlying math-gender stereotypes. So far, math-gender stereotypes have been assessed either via implicit association testing (e.g., Nosek et al., 2010; Steffens and Jelenec, 2011), or ratings of whether math is more female than male (e.g., Nosek et al., 2010), or via one to three simple items about talent differences (see Hyde et al., 1990; Gunderson et al., 2012). The current assessment of math-gender misconceptions as a construct underlying math-gender stereotypes is a novel approach to understand and potentially refute math-gender gender stereotypes. In the future, assessing math-gender misconceptions in addition to math-gender stereotypes may facilitate the comprehension of math-gender stereotypes and thus our ability to target both – math-gender stereotypes and math-gender misconceptions. With this reasoning, it is important to note that holding the math-gender stereotype correlated most strongly with holding the *empathizing-systemizing* and the *girls’ compensation* misconception. Specifically targeting these misconceptions (e.g., by means of refutation text; Tippett, 2010) may thus be a promising means to reduce not just the specific misconception but also math-gender stereotypes to a certain degree. More research applying more measures for math-gender stereotypes and evaluating their association with math-gender misconceptions is necessary to gain more insights into the association between math-gender misconceptions, implicit and explicit math-gender stereotypes, as well as how they manifest in teacher and student teacher behavior. Additionally, applying more measures of explicit math-gender stereotypes in future research to assess the relations between math-gender stereotypes and math-gender misconceptions should help further validate the MGMQ in future research. Hence, this study is the first of a planned series of studies on the relationship between math-gender misconceptions and math-gender stereotypes.

### Limitations and Further Research

In this study, we presented the MGMQ, a novel measure assessing misconceptions about gender differences in math abilities. To the best of our knowledge, this is the first study applying the concept of misconceptions (e.g., Eitel et al., 2019) to the important field of women’s underrepresentation in math. Therefore, some limitations and questions for future research emerged.

First of all, as the main objective of this research was the construction and evaluation of the MGMQ, we implemented only *one* measure to assess math-gender stereotypes [similarly applied by Nosek et al. (2010)]. It is certainly useful to relate the MGMQ results to other measures assessing math-gender stereotypes in further research. In this paper, we described the MGMQ development. As the MGMQ has demonstrated its reliability as a measuring tool within our sample, we intend to further research its reliability and interrelations between the MGMQ scales and various implicit and explicit – as well as behavioral stereotype-measures in future research.

Another limitation refers to the risk of triggering socially desirable responses as discussed before. Furthermore, recognizing and reporting socially undesirable stereotypes may require a certain degree of self-awareness among participants (Nosek, 2007). Some may not have thought about their stereotypes because they were unwilling to. But even though reflection is necessary and social desirability may hinder the readiness to self-report stereotypes, direct self-reporting is still known to work best for assessing stereotypes (Axt, 2018). In future research, some items (e.g., “Since girls are on average less mathematically gifted, they should be assessed with different criteria than boys”) could be revised to make them more neutral-sounding. Strongly overlapping items could be excluded, forming a short version of the MGMQ (e.g., “As girls think more empathically whereas boys think more systematically, boys are on average more talented for math than girls”). A short version should be economic and especially practical for applying it to in-service teachers, as they have less time to participate in research. In future studies, it would be also promising for researchers to stress that the MGMQ is a knowledge test, not an attitude test, thus hopefully reducing further answer bias due to social desirability. Future research with the MGMQ could also focus on the prevalence of math-gender misconceptions in math teachers, as math teachers, due to their direct influence on girls’ math learning, may contribute especially to the upholding of math-gender misconceptions (and math-gender stereotypes). In this regard, we compared the misconception prevalence between student teachers with and without mathematics as teaching subjects here. We observed small and insignificant differences between students with math (*M* = −1.64, *SD* = 1.17) and without math as teaching subject (*M* = −1.48, *SD* = 1.40), *t*(301) = 1.04, *p* = 0.30.

Further, our sample’s gender distribution consisting of 79.9% women does not represent the general population. However, this high percentage of women in our student teacher sample resembles the gender distribution of teachers in Germany: The Federal Office for Statistics in Germany assessed teachers’ gender in the school year of 2019/2020 and found that 73.1% of teachers in general education were female. As gender might still have influenced the math-gender misconception prevalence, we compared the prevalence rates between genders, and revealed that the prevalence of math-gender misconceptions did not differ between female participants (*M* = −1.56, *SD* = 1.33) and male participants (*M* = −1.46, *SD* = 1.27), *t*(301) = 0.54, *p* = 0.58. This insignificant difference may be due to the exposure to math-gender misconceptions in our society regardless of gender.

Furthermore, implicit and behavioral measures could support the assessment and generate additional knowledge about the prevalence of math-gender misconceptions or math-gender stereotypes.

The goal of the MGMQ is to identify math-gender misconceptions that potentially underlie math-gender stereotypes. As (math-gender) stereotypes have rarely been successfully reduced (FitzGerald et al., 2019; Kollmayer et al., 2020), identifying underlying math-gender misconceptions is a starting point for conceptual change – and hopefully attitude change as well. Interventions targeting misconceptions among teachers (e.g., refutation texts; Menz et al., 2021) could therefore also be applied to revise or reduce stereotypes among teachers.

In addition to the math-gender misconceptions discussed here, there are misconceptions and ideas associated with other stereotypes that influence math representation and warrant research. This should yield insights on whether such associations between stereotypes and misconceptions are specific to the gender topic, or generalizable. One example would be math-race stereotypes (Starr and Simpkins, 2021). The intersectionality of stereotypes, meaning people belonging to more than one minority group (e.g., Black and female) and thus suffering from different overlapping adverse stereotypes, should be considered in future research (Yuval-Davis, 2006; Parker et al., 2020).

### Conclusion

This study describes a newly developed instrument assessing misconceptions about gender differences in math ability that potentially underlie gender stereotypes, and which therefore may contribute to the underrepresentation of women in math careers. Our results show that (a) our newly developed questionnaire reliably assessed three distinct misconceptions related to gender differences in mathematics in the first sample, (b) almost half of the participating preservice teachers endorsed at least one of the three misconceptions, whereas a majority did not, and (c) holding these misconceptions was substantially associated with holding math-gender stereotypes.

Identifying the specific misconceptions potentially behind math-gender stereotypes is a good starting point for interventions aiming at conceptual change (Larkin, 2012), also in the field of gender and STEM. Since misconceptions hinder the acquisition of scientifically accurate conceptions (Eitel et al., 2021), overcoming them is important to reduce gender disparities in STEM in the future. This study provides the basis upon which to develop specific instructions in the form of refutation texts during teacher education or training (Eitel et al., 2019; Menz et al., 2021; Dersch et al., 2022).

## Data Availability Statement

The raw data supporting the conclusions of this article will be made available by the authors, without undue reservation.

## Ethics Statement

The studies involving human participants were reviewed and approved by the Lokale Ethik-Kommission des Fachbereichs 06 der Justus-Liebig-Universität Gießen. The patients/participants provided their written informed consent to participate in this study.

## Author Contributions

A-SD, AH, and AE contributed to the conception and design of the study and wrote the sections of the manuscript. A-SD organized the database and wrote the first draft of the manuscript. A-SD and AE performed the statistical analysis. All authors contributed to manuscript revision, read, and approved the submitted version.

## Conflict of Interest

The authors declare that the research was conducted in the absence of any commercial or financial relationships that could be construed as a potential conflict of interest.

## Publisher’s Note

All claims expressed in this article are solely those of the authors and do not necessarily represent those of their affiliated organizations, or those of the publisher, the editors and the reviewers. Any product that may be evaluated in this article, or claim that may be made by its manufacturer, is not guaranteed or endorsed by the publisher.
