# Supplementary material for: Exploring the Nature of Teachers’ Math-Gender Stereotypes: The Math-Gender Misconception Questionnaire
Source: Front Psychol. 2022 Apr 14;13:820254. doi: 10.3389/fpsyg.2022.820254 (PMC9046592; doi:10.3389/fpsyg.2022.820254)
Supplement: Supplementary file 1 [file Data_Sheet_1.pdf]

Supplementary Material

Appendix A

Full German Original of the Math-Gender Misconception Questionnaire

Im Folgenden werden Sie verschiedene Aussagen zu Mathematik und Geschlecht lesen. Bitte geben Sie jeweils an, ob Sie der Aussage zustimmen und wie sicher Sie sich in Ihrer Einschätzung sind.

Wir bitten Sie auf längere Pausen während der 10-minütigen Bearbeitung zu verzichten. Bitte beachten Sie, dass es während der gesamten Studie keinen *zurück*-Button gibt. Bitte klicken Sie auch im Browser nicht auf *zurück*, um zur vorherigen Seite zu gelangen. Sollten Sie eine oder mehrere Fragen vergessen, werden Sie vor dem weiter-Klicken in roter Schrift daran erinnert, diese zu beantworten.

Das Schulfach Mathematik stößt bei Mädchen häufiger auf Ablehnung als bei Jungen.

- ○ stimme nicht zu
- ○ stimme zu

Wie sicher sind Sie sich bezüglich dieser Aussage?

- ○ sehr unsicher
- ○ unsicher
- ○ etwas sicher
- ○ ziemlich sicher
- ○ sehr sicher

Da Mädchen eher empathisch denken und Jungen eher systematisch denken, sind Jungen durchschnittlich begabter für Mathematik als Mädchen.

- ○ stimme nicht zu
- ○ stimme zu

Wie sicher sind Sie sich bezüglich dieser Aussage?

- ○ sehr unsicher
- ○ unsicher
- ○ etwas sicher
- ○ ziemlich sicher
- ○ sehr sicher

Jungen fliegen mathematische Inhalte häufig einfach zu, während Mädchen sich dafür durchschnittlich mehr anstrengen müssen.

- stimme nicht zu
- stimme zu

Wie sicher sind Sie sich bezüglich dieser Aussage?

- sehr unsicher
- unsicher
- etwas sicher
- ziemlich sicher
- sehr sicher

Da Mädchen durchschnittlich eine geringere mathematische Begabung mitbringen, sollten sie anhand anderer Kriterien bewertet werden als Jungen.

- stimme nicht zu
- stimme zu

Wie sicher sind Sie sich bezüglich dieser Aussage?

- sehr unsicher
- unsicher
- etwas sicher
- ziemlich sicher
- sehr sicher

Bei Mädchen und Jungen sind während der Bearbeitung von Rechenaufgaben ähnliche Hirnregionen aktiv.

- stimme nicht zu
- stimme zu

Wie sicher sind Sie sich bezüglich dieser Aussage?

- sehr unsicher
- unsicher
- etwas sicher
- ziemlich sicher
- sehr sicher

Mathematische Zusammenhänge erschließen sich für Jungen im Normalfall leichter als für Mädchen, da Jungen eher in systematischen Zusammenhängen denken.

- ○ stimme nicht zu
- ○ stimme zu

Wie sicher sind Sie sich bezüglich dieser Aussage?

- ○ sehr unsicher
- ○ unsicher
- ○ etwas sicher
- ○ ziemlich sicher
- ○ sehr sicher

Bereits im Grundschulalter denken Schüler*innen, dass mathematische Berufe eher etwas für Männer sind.

- ○ stimme nicht zu
- ○ stimme zu

Wie sicher sind Sie sich bezüglich dieser Aussage?

- ◉ sehr unsicher
- ○ unsicher
- ○ etwas sicher
- ○ ziemlich sicher
- ○ sehr sicher

Mädchen müssen sich im Normalfall mehr anstrengen, um die gleiche Leistung in Mathematik zu erbringen wie Jungen.

- ○ stimme nicht zu
- ○ stimme zu

Wie sicher sind Sie sich bezüglich dieser Aussage?

- ○ sehr unsicher
- ○ unsicher
- ○ etwas sicher
- ○ ziemlich sicher
- ○ sehr sicher

Mädchen gleichen ihre durchschnittlich geringere Begabung im Fach Mathematik im Vergleich zu Jungen durch mehr Fleiß aus.

- stimme nicht zu
- stimme zu

Wie sicher sind Sie sich bezüglich dieser Aussage?

- sehr unsicher
- unsicher
- etwas sicher
- ziemlich sicher
- sehr sicher

Mädchen sollten für ihre stärkeren Anstrengungen im Fach Mathematik mit guten Noten belohnt werden, da sie von Natur aus nicht so gut in Mathematik sind wie Jungen.

- stimme nicht zu
- stimme zu

Wie sicher sind Sie sich bezüglich dieser Aussage?

- sehr unsicher
- unsicher
- etwas sicher
- ziemlich sicher
- sehr sicher

Jungen zeigen häufiger Begeisterung für Mathematik als Mädchen.

- stimme nicht zu
- stimme zu

Wie sicher sind Sie sich bezüglich dieser Aussage?

- sehr unsicher
- unsicher
- etwas sicher
- ziemlich sicher
- sehr sicher

Mädchen würden im Mathematikunterricht motiviert, wenn mehr weiblich konnotierte Themen Inhalt der Aufgaben und des Unterrichts wären.

- stimme nicht zu
- stimme zu

Wie sicher sind Sie sich bezüglich dieser Aussage?

- ○ sehr unsicher
- ○ unsicher
- ○ etwas sicher
- ○ ziemlich sicher
- ○ sehr sicher

Weil Jungen eher in systematischen Kategorien denken, haben sie in Mathematik bessere Voraussetzungen als Mädchen.

- ○ stimme nicht zu
- ○ stimme zu

Wie sicher sind Sie sich bezüglich dieser Aussage?

- ○ sehr unsicher
- ○ unsicher
- ○ etwas sicher
- ○ ziemlich sicher
- ○ sehr sicher

Im Durchschnitt schätzen sich Jungen in Mathematik als kompetenter ein als Mädchen.

- ○ stimme nicht zu
- ○ stimme zu

Wie sicher sind Sie sich bezüglich dieser Aussage?

- ○ sehr unsicher
- ○ unsicher
- ○ etwas sicher
- ○ ziemlich sicher
- ○ sehr sicher

Mathematiklehrkräfte schätzen Mathematik im Durchschnitt als eine eher männliche Domäne ein.

- ○ stimme nicht zu
- ○ stimme zu

Wie sicher sind Sie sich bezüglich dieser Aussage?

- ◉ sehr unsicher

- unsicher
- etwas sicher
- ziemlich sicher
- sehr sicher

Die Verwendung männlich konnotierter Beispiele in Mathematikaufgaben wirkt auf Mädchen demotivierend.

- stimme nicht zu
- stimme zu

Wie sicher sind Sie sich bezüglich dieser Aussage?

- sehr unsicher
- unsicher
- etwas sicher
- ziemlich sicher
- sehr sicher

Mädchen benötigen üblicherweise zusätzliche Förderung, um gleichwertige Leistungen im Fach Mathematik zu erbringen wie Jungen.

- stimme nicht zu
- stimme zu

Wie sicher sind Sie sich bezüglich dieser Aussage?

- sehr unsicher
- unsicher
- etwas sicher
- ziemlich sicher
- sehr sicher

Ist der Klassenbeste in Mathematik ein Junge, liegt das daran, dass er zusätzlich zu seiner Anstrengung ein natürliches mathematisches Talent mitbringt, welches fleißigen Mädchen fehlt.

- stimme nicht zu
- stimme zu

Wie sicher sind Sie sich bezüglich dieser Aussage?

- sehr unsicher
- unsicher
- etwas sicher
- ziemlich sicher

- sehr sicher

Die weibliche Empathie erleichtert Mädchen den Umgang mit Menschen, während Jungen durchschnittlich begabter im systematischen Denken und somit in Mathematik sind.

- stimme nicht zu
- stimme zu

Wie sicher sind Sie sich bezüglich dieser Aussage?

- sehr unsicher
- unsicher
- etwas sicher
- ziemlich sicher
- sehr sicher

Unter anderem aufgrund ihres systematischen Denkens sind Jungen durchschnittlich interessierter an Mathematik als Mädchen.

- stimme nicht zu
- stimme zu

Wie sicher sind Sie sich bezüglich dieser Aussage?

- [x] sehr unsicher
- unsicher
- etwas sicher
- ziemlich sicher
- sehr sicher

Im Durchschnitt haben Mädchen in der Oberstufe etwa gleich gute Mathematiknoten wie Jungen.

- stimme nicht zu
- stimme zu

Wie sicher sind Sie sich bezüglich dieser Aussage?

- sehr unsicher
- unsicher
- etwas sicher
- ziemlich sicher
- sehr sicher

Für gleich gute Noten in Mathematik müssen sich Jungen weniger anstrengen, da sie mehr Talent mitbringen als Mädchen.

- ○ stimme nicht zu
- ○ stimme zu

Wie sicher sind Sie sich bezüglich dieser Aussage?

- ○ sehr unsicher
- ○ unsicher
- ○ etwas sicher
- ○ ziemlich sicher
- ○ sehr sicher

Selbst Mädchen, die sehr gute Noten in Mathematik haben, ergreifen selten mathematische Berufe.

- ○ stimme nicht zu
- ○ stimme zu

Wie sicher sind Sie sich bezüglich dieser Aussage?

- ○ sehr unsicher
- ○ unsicher
- ○ etwas sicher
- ○ ziemlich sicher
- ○ sehr sicher

Jungen sind durchschnittlich motivierter für Mathematik als Mädchen.

- ○ stimme nicht zu
- ○ stimme zu

Wie sicher sind Sie sich bezüglich dieser Aussage?

- ○ sehr unsicher
- ○ unsicher
- ○ etwas sicher
- ○ ziemlich sicher
- ○ sehr sicher

Mädchen denken im Durchschnitt empathischer als Jungen, dafür sind Jungen talentierter in systematischem Denken und somit auch in Mathematik.

- ○ stimme nicht zu
- ○ stimme zu

Wie sicher sind Sie sich bezüglich dieser Aussage?

- sehr unsicher
- unsicher
- etwas sicher
- ziemlich sicher
- sehr sicher

Ihre fehlende Veranlagung für Mathematik können Mädchen mit ihrem durchschnittlich höheren Fleiß nicht ganz ausgleichen.

- stimme nicht zu
- stimme zu

Wie sicher sind Sie sich bezüglich dieser Aussage?

- sehr unsicher
- unsicher
- etwas sicher
- ziemlich sicher
- sehr sicher

Jungen identifizieren sich stärker mit dem Fach Mathematik als Mädchen.

- stimme nicht zu
- stimme zu

Wie sicher sind Sie sich bezüglich dieser Aussage?

- sehr unsicher
- unsicher
- etwas sicher
- ziemlich sicher
- sehr sicher

In der Identitätsbildung wenden sich Mädchen häufig vom Fach Mathematik ab, um sich so stärker der Gruppe der Frauen zugehörig zu fühlen.

- stimme nicht zu
- stimme zu

Wie sicher sind Sie sich bezüglich dieser Aussage?

- sehr unsicher
- unsicher
- etwas sicher
- ziemlich sicher
- sehr sicher

Trotz ihrer durchschnittlich stärkeren Anstrengung sind Mädchen in Mathematik im Normalfall weniger gut als Jungen.

- stimme nicht zu
- stimme zu

Wie sicher sind Sie sich bezüglich dieser Aussage?

- sehr unsicher
- unsicher
- etwas sicher
- ziemlich sicher
- sehr sicher

Werden Mädchen in Mathematik stärker gelobt als Jungen, kann dies für sie demotivierend sein.

- stimme nicht zu
- stimme zu

Wie sicher sind Sie sich bezüglich dieser Aussage?

- sehr unsicher
- unsicher
- etwas sicher
- ziemlich sicher
- sehr sicher

Appendix B

German and English Items of the Assessment of Feminism Amongst the Participants.

German original	English translation
**Wie wichtig ist Ihnen die Gleichberechtigung der Geschlechter?**<br><br>Gar nicht wichtig<br><br>Nicht so wichtig<br><br>Etwas wichtig<br><br>Ziemlich wichtig<br><br>Sehr wichtig	**How important is the equality of the genders to you?**<br><br>Not at all important<br><br>Not so important<br><br>Somewhat important<br><br>Quite important<br><br>Very important
**Setzen Sie sich mit Feminismus auseinander?**<br><br>Gar nicht<br><br>Nicht sonderlich<br><br>Etwas<br><br>Ziemlich<br><br>Sehr stark	**Do you engage with feminism?**<br><br>Not at all<br><br>Not particularly<br><br>Somewhat<br><br>Quite somewhat<br><br>Very much
**Würden Sie sich selbst als Feminist*in bezeichnen?**<br><br>Gar nicht<br><br>Nicht wirklich<br><br>Etwas<br><br>Ziemlich<br><br>Absolut	**Would you consider yourself a feminist?**<br><br>Not at all<br><br>Not really<br><br>Somewhat<br><br>Quite somewhat<br><br>Absolutely
